# Single-Channel Sleep EEG Classification Method Based on LSTM and Hidden Markov Model

**DOI:** 10.3390/brainsci14111087

**Published:** 2024-10-29

**Authors:** Wan Chen, Yanping Cai, Aihua Li, Yanzhao Su, Ke Jiang

**Affiliations:** School of Combat Support, Rocket Force University of Engineering, Xi’an 710025, China; chenwan305@126.com (W.C.); l863@163.com (A.L.); syzlhh@163.com (Y.S.); jugglerchn@foxmail.com (K.J.)

**Keywords:** single-channel EEG, long short-term memory, classification of sleep stages, wavelet transform, hidden Markov model

## Abstract

Background: The single-channel sleep EEG has the advantages of convenient collection, high-cost performance, and easy daily use, and it has been widely used in the classification of sleep stages. Methods: This paper proposes a single-channel sleep EEG classification method based on long short-term memory and a hidden Markov model (LSTM-HMM). First, the single-channel EEG is decomposed using wavelet transform (WT), and multi-domain features are extracted from the component signals to characterize the EEG characteristics fully. Considering the temporal nature of sleep stage changes, this paper uses a multi-step time series as the input for the model. After that, the multi-step time series features are input into the LSTM. Finally, the HMM improves the classification results, and the final prediction results are obtained. Results: A complete experiment was conducted on the Sleep-EDFx dataset. The results show that the proposed method can extract deep information from EEG and make full use of the sleep stage transition rule. The proposed method shows the best performance in single-channel sleep EEG classification; the accuracy, macro average F1 score, and kappa are 82.71%, 0.75, and 0.76, respectively. Conclusions: The proposed method can realize single-channel sleep EEG classification and provide a reference for other EEG classifications.

## 1. Introduction

Classification of sleep stages is essential for monitoring sleep quality and diagnosing related sleep disorders [1]. The American Academy of Sleep Medicine (AASM) divides sleep stages into five stages, namely wakefulness (W), rapid eye movement (REM), and non-REM (subdivided into N1, N2, and N3) [2]. Experts usually use polysomnography (PSG) to classify sleep stages. PSG includes electroencephalogram (EEG), electrooculogram (EOG), electromyogram (EMG), and electrocardiogram (ECG) [3]. Manual recognition of PSG takes time and effort. In addition, it is not easy to collect PSG, which is not conducive to daily family use [4]. Single-channel EEG signals are easy to collect, convenient for daily family use, and have received more attention in classifying sleep stages [5]. Therefore, proposing an automatic sleep stage classification method based on single-channel sleep EEG is significant.

Feature extraction and classification of single-channel EEG is the key to the automatic classification of sleep stages [6,7,8]. Venkat and colleagues [9] used wavelet packet decomposition to extract five sub-bands from EEG and then extracted Hjorth parameters and feature ratios between different bands from the sub-bands. Finally, K-nearest neighbor (KNN) and SVM were used to classify the sleep stages. Liu and colleagues [10] used ensemble empirical mode decomposition (EEMD) to decompose single-channel sleep EEG and then extracted time-domain features and nonlinear features from each intrinsic modal component (IMF). Jiang and colleagues [11] used empirical mode decomposition (EMD) to decompose single-channel EEG and then extracted multiple time domains, frequency domains, and nonlinear features from the first seven IMFs. Finally, RF was used to realize automatic sleep stage classification. The above studies used signal decomposition for feature extraction, which can fully utilize EEG information. However, the existing studies lacked comparative studies on different signal decomposition methods.

In recent years, more and more studies have shown that deep learning performs well in sleep EEG classification [5,12]. Sharma and colleagues [13] used a wavelet-scattering network to extract EEG features from a single-channel EEG and used a weighted K-nearest neighbor algorithm (WKNN) to classify sleep stages. Phan and colleagues [14] proposed a joint classification and prediction framework based on convolutional neural networks (CNN) and adopted a one-to-many classification strategy to realize automatic sleep EEG classification. Heng and colleagues [15] pointed out that the CNN could extract the time–frequency features of EEG signals, and the gated recurrent unit (GRU) could learn the transition rule of sleep stages. They built an end-to-end network based on CNN and GRU to achieve single-channel sleep EEG classification. Considering the potential regularity of sleep state transition, some studies have begun to use networks with temporal information-learning ability to classify single-channel sleep EEG, including long short-term memory (LSTM) and transformer [16]. LSTM can capture the timing information of signals and excels in processing sequential data with long-term dependencies [16]. The transformer network can learn contextual information, offering advantages in handling short sequences and enabling parallel computation [1]. Nevertheless, most existing studies primarily rely on single-step time data as the input for their models, which hampers the models’ capacity to effectively learn the transition rules between different sleep stages [17,18].

The method based on temporal networks can learn the sleep transition rules during the classification process and improve accuracy. However, due to the classifier’s limited performance, some misclassification is inevitable. According to the previous sleep state, some misidentified sleep states can be well corrected. Ghimatgar and colleagues [19] proposed a single-channel sleep EEG classification method based on the random forest (RF) and hidden Markov model (HMM). First, RF is employed to classify sleep EEG, and then, the HMM is used to learn the sleep transition rules. The experimental results validate the effectiveness of this approach. Networks such as LSTM and Transformer [1,18] are capable of learning sleep stage transition rules during classification, whereas HMM [11] focuses on understanding these transition rules post-classification. Consequently, this paper explores the combination of a temporal network with HMM to learn the sleep transition rules from both perspectives, which has the potential to enhance the classification accuracy of single-channel sleep EEG.

This paper proposed a single-channel sleep EEG classification method based on LSTM and HMM (LSTM-HMM). The contributions of this paper are as follows.

(1) We compared the performances of EMD, VMD, SSA, and WT in the decomposition of single-channel sleep EEG. Further, we analyzed the performance of twenty wavelet functions, which provided a reference for other researchers for EEG decomposition and feature extraction;

(2) The proposed method considered the temporal structure of the sleep stage transition from two perspectives. First, the multi-step time features and LSTM were used to learn the sleep transition rules during the classification. After classification, the HMM was used to find the most likely sleep state transition sequence and automatically corrected the results;

(3) The proposed method was fully verified on the Sleep-EDFx dataset. The results show that WT can extract deep information from EEG. The proposed method can achieve high-precision sleep EEG classification using the sleep stage transition rules and is superior to most existing methods.

## 2. Method

**Figure 1:**
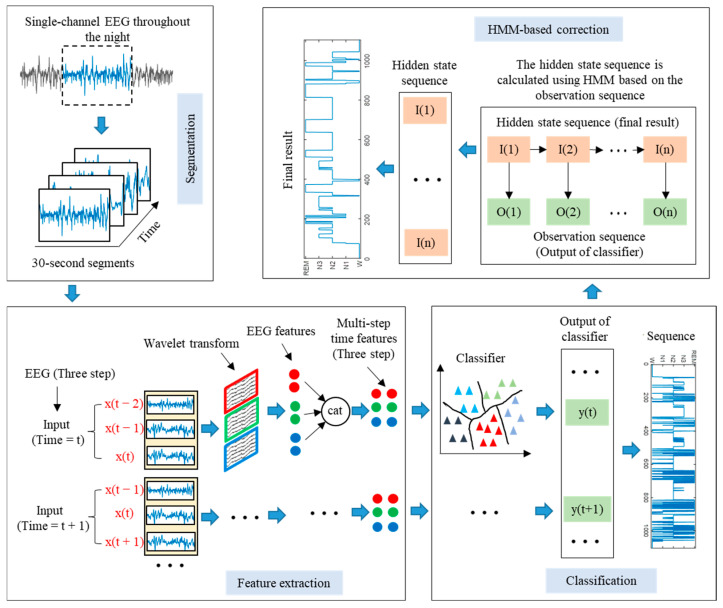
Framework of proposed method.

The single-channel EEG classification method proposed in this paper based on LSTM-HMM is shown in Figure 1. The proposed method can be divided into four stages, namely EEG segmentation, EEG feature extraction, EEG classification, and HMM-based correction. First, the entire night’s sleep EEG signals were segmented into 30 s segments. Then, the EEG was decomposed by WT, and the time domain, frequency domain, and nonlinear feature were extracted. After that, the multi-step time features were input into the LSTM network to realize sleep EEG classification. Finally, the predicted sleep state sequence throughout the night was input into the HMM, and the hidden state sequence with the most significant probability was obtained, which is the final prediction result.

### 2.1. EEG Decomposition Based on Wavelet Transform

**Figure 2:**
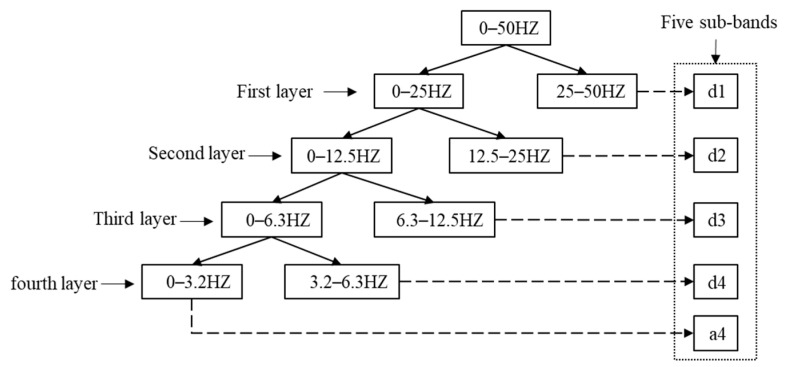
Sub-band distribution of EEG decomposed with four-layer DWT.

The spatial resolution of the single-channel EEG is low, and the information obtained directly is small. Before feature extraction, this paper uses the decomposition method to decompose single-channel EEG signals to extract more hidden information. WT is a time–frequency decomposition positioning technique that decomposes signals through stretched and shifted wavelet functions [20]. Compared with EMD, VMD, and SSA, WT can provide better time–frequency positioning [21]. In addition, the most helpful information in sleep EEG is concentrated in low-frequency components below 30 HZ, and WT has significant advantages in extracting accurate low-frequency data [22]. This paper uses discrete wavelet transform (DWT) to decompose the single-channel EEG signal. The DWT is defined as follows:

(1) $$WT_{f}(j,k)=\int f(t)\varphi_{j,k}(t)dt$$

(2) $$\varphi_{j,k}(t)=2^{\frac{-j}{2}}\varphi (2^{-j}t-k)$$

where f(t) represents the signal and $\varphi_{j,k}(t)$ represents the wavelet function. j and k represent the scale and displacement parameters. Using *k*-layer DWT to decompose the single-channel EEG x(t), the *k* + 1 sub-bands are obtained.

(3) $$x(t)=a_{k}(t)+\sum_{l=1}^{k}d_{l}(t)$$

where $a_{k}(t)$ and $d_{l}(t)$ represent the low-frequency and high-frequency components of the EEG. The EEG is usually divided into five rhythmic waves; the lowest frequency rhythmic wave is delta (0–4 HZ). In order to excavate the hidden information of the EEG as much as possible, avoid excessive decomposition layers to increase the computing load. The signal decomposition is complete when the low-frequency component $a_{k}(t)$ is in the delta. In this paper, the Sleep-EDFx dataset is used for the experiments. The sampling frequency of the EEG is 100 HZ, so a four-layer DWT is designed to decompose the EEG. The distribution of sub-bands is shown in Figure 2.

Different wavelet functions (WF) can affect the decomposition results of EEG [21,23]. This paper analyzed the performance of 20 different wavelet functions in EEG decomposition, which can provide a reference for other EEG-related research. The detailed information on the WF is shown in Table 1.

### 2.2. Time Domain, Frequency Domain, and Nonlinear Feature Extraction

The single-channel EEG signal was decomposed to obtain five sub-bands, as shown in Figure 2. After that, multiple time domains, frequency domains, and nonlinear features are extracted from the five sub-bands to explore the EEG information fully. The EEG features extracted in this paper are shown in Table 2. For an EEG x with length N, the specific calculation of feature extraction is as follows.

#### 2.2.1. Time Domain Features

Time domain features can provide the characteristics of signals in the time domain [9]. The time-domain features extracted in this paper can be divided into statistical and Hjorth parameters. The statistical parameters include the absolute mean value (*MA*), standard deviation (*Std*), skewness (*Ske*), and kurtosis (*Kur*) of EEG [10].

(4) $$MA(x)=\frac{1}{N}\sum_{i=1}^{N}\left|x_{i}\right|$$

(5) $$Std(x)=\sqrt{\frac{1}{N}\sum_{i=1}^{N}{\left(x_{i}-\bar{x}\right)}^{2}}$$

(6) $$Ske(x)=\frac{1}{N}\sum_{i=1}^{N}{\left(\frac{x_{i}-\bar{x}}{x_{std}}\right)}^{3}$$

(7) $$Kur(x)=\frac{1}{N}\sum_{i=1}^{N}{\left(\frac{x_{i}-\bar{x}}{x_{std}}\right)}^{4}$$

Hjorth parameters measure the characteristics of signals in the time domain from three aspects, namely activity (*HA*), mobility (*HM*), and complexity (*HC*).

(8) $$HA(x)=\frac{1}{N}\sum_{i=1}^{N}{\left(x_{i}-\bar{x}\right)}^{2}$$

(9) $$HM(x)=\sqrt{\frac{HA(dx/dt)}{HA(x)}}$$

(10) $$HC(x)=\frac{HM(dx/dt)}{HM(x)}$$

#### 2.2.2. Frequency Domain Features

Frequency domain analysis requires the conversion of the EEG from the time domain to the frequency domain. This paper converts the time domain EEG signal into the frequency domain with the fast Fourier transform [24]. After that, the statistical parameters of the signal in the frequency domain are extracted, namely the mean, standard deviation, skewness, kurtosis, and mean square value. In addition, the power spectral density (*PSD*) of the signal is calculated, and the average power spectral density (*Mpsd*) and power (*P*) are extracted from the *PSD* [16].

(11) $$Mpsd=\frac{1}{N}\sum_{i=1}^{N}PSD_{i}$$

(12) $$P=\int_{w}PSD(w)dw$$

where w represents frequency. In addition, the power ratio between five sub-bands is extracted: $P_{d1/d2}$, $P_{d1/d3}$, $P_{d1/d4}$, $P_{d1/a4}$, $P_{d2/d3}$, $P_{d2/d4}$, $P_{d2/a4}$, $P_{d3/d4}$, $P_{d3/a4}$, $P_{d4/a4}$.

#### 2.2.3. Nonlinear Features

An EEG is a typical nonlinear signal, so the nonlinear features can measure the nonlinearity of EEG [23]. This paper extracted five nonlinear features, namely approximate entropy (*AE*), differential entropy (*DE*), Shannon entropy (*SE*), CO complexity (*CC*), and fractal dimension (*FD*) [10].

(1) Approximate entropy (*AE*)

*AE* is used to quantify the regularity and unpredictability of signal fluctuations [24]. First, the m-dimension reconstruction of EEG x is carried out:

(13) $$Y_{m}(i)=\{x(i),x(i+1),\cdots ,x(i+m-1)\},1⩽i⩽N-m+1$$

Define the distance between $Y_{m}(i)$ and $Y_{m}(j)$ as:

(14) $$d[Y_{m}(i),Y_{m}(j)]=\underset{k=0,\cdots ,m-1}{\max}(|x(i+k)-x(j+k)|)$$

Given the threshold r, count the number of d≤r:

(15) $$B_{i}^{m}(r)=\frac{N_{d\leq r}^{i}}{N-m-1}$$

Define $\Phi^{m}(r)$ as:

(16) $$\Phi^{m}(r)={\left(N-m+1\right)}^{-1}\overset{N-m+1}{\underset{i=1}{\sum }}\log(B_{i}^{m}(r))$$

Finally, the *AE* of the EEG is obtained:

(17) $$AE=\Phi^{m}(r)-\Phi^{m+1}(r)$$

(2) Shannon entropy (*SE*)

*SE* is used to measure the uncertainty ratio of a signal [25]. The greater the *SE*, the greater the randomness of the signal. *SE* is defined as:

(18) $$SE=-\sum_{i=1}^{n}p(x_{i})\log(p(x_{i}))$$

where $p(x_{i})$ represents the probability of the occurrence of a random event $x_{i}$.

(3) Differential entropy (*DE*)

*DE* is a generalization of Shannon entropy on continuous variables. EEG approximately follows a Gaussian distribution $N(\mu ,\sigma^{2})$, and its *DE* is [26]:

(19) $$DE=-\int_{x}p(x)\log(p(x))dx=\frac{1}{2}\log(2\pi \sigma^{2})$$

(4) CO complexity (*CC*)

*CC* is used to measure the degree of irregularity of the signal [27]. First, Fourier transforms the signal and calculates the average value of the power spectrum:

(20) $$M=\frac{1}{N}\sum_{i=1}^{N}|X_{i}{|}^{2}$$

where X represents the result of the Fourier transform of EEG. After that, define a new sequence:

(21) $$Y=\left\{\begin{array}{l} X\;|X{|}^{2}>M\\ 0\;|X{|}^{2}\leq M \end{array}\right.$$

Finally, the CO complexity of EEG is obtained:

(22) $$CC=\frac{\sum_{i=1}^{N}|x_{i}-y_{i}{|}^{2}}{\sum_{i=1}^{N}|x_{i}{|}^{2}}$$

where y represents the inverse Fourier transform result of Y.

(5) Fractal dimension (*FD*)

The *FD* measures the complexity of a signal from the perspective of chaotic dynamics. In this paper, the Higuchi method is used to calculate the *FD* [11]. First, the signal is converted into τ sequences:

(23) $$X_{\tau }^{T}=\{x(\tau ),x(\tau +T),\ldots ,x(\tau +[\frac{N-\tau }{T}])\}$$

where τ=1,2,…,T. Define the length of each sequence as:

(24) $$L_{\tau }(T)=\frac{1}{T}\frac{\sum_{i=1}^{\left[\frac{N-\tau }{T}\right]}|x(\tau -\mathrm{iT})-x(\tau (i-1)T)|\times (N-1)}{\left[\frac{N-\tau }{T}\right]}$$

After that, the average of each sequence length is calculated:

(25) $$L(T)=\overset{T}{\underset{\tau =1}{\sum }}L_{\tau }(T)$$

Given the interval $\left[T_{\min},T_{\max}\right]$, calculate the corresponding L(T). lnL(T) and ln(1/T) are fitted linearly, and the slope of the linear fitting is the fractal dimension of EEG.

### 2.3. Classifier

The extracted features are reconstructed into multi-step time features and input into the classifier to realize sleep EEG classification. Ten classifiers are used in this paper, namely radial basis function support vector machine (RBFSVM), linear function support vector machine (LFSVM), random forest (RF), decision tree (DT), naive Bayes (NB), K-nearest neighbor (KNN), convolutional neural network (CNN), long short-term memory (LSTM), bidirectional LSTM (Bi-LSTM), and transformer encoder (TE).

(1) Support vector machine

SVM is a machine-learning method based on statistical learning theory, which performs well for small sample data [28]. The core idea of SVM is to construct an optimal hyperplane in the projection space, separate different types of data, and maximize the distance between the two types of data [23]. Different kernel functions will affect the performance of SVM. This paper uses the radial basis function and linear function as kernel functions:

(26) $$K_{RBF}(x,z)=\exp(-\frac{{\left\|x-z\right\|}^{2}}{2\sigma^{2}})$$

(27) $$K_{LF}(x,z)=x^{T}z$$

where σ represents the bandwidth of the kernel function. The penalty factor and kernel bandwidth of the support vector machine are set to C=1 and σ=0.01, respectively.

(2) Random forest

RF is an ensemble-learning model based on the Bagging strategy, which has better robustness to noise, lower complexity, and faster computing speed [22]. There are many classification trees in the RF. Each classification tree classifies the samples during decision-making and determines the sample category according to the voting results [19]. The two cores of RF are sample randomness and feature randomness [12]. Sample randomization refers to sampling some samples randomly from the original dataset to form some sub-datasets. Feature randomization means that, when selecting the optimal feature, only the subset of features selected randomly is considered rather than all of the features. The RF’s tree number and depth are set to 60 and 10, respectively, and the classification tree is constructed using the C4.5 algorithm.

(3) Decision tree

DT is a model that displays decision rules and classification results with a tree-like data structure [23]. The DT consists of a root node and several internal and several leaf nodes. Each internal node represents a test of a feature attribute. Each branch represents the test result, and each leaf node represents the decision result. In this paper, the C4.5 algorithm is used to generate the DT. The C4.5 algorithm uses the information gain ratio to discretize continuous features, thus achieving feature selection [29]. The number depth of the DT is set to 10.

(4) Naive Bayes

NB is a classification method based on Bayes’ theorem and feature independence assumption [30]. The core idea of NB is to use Bayes’ theorem to calculate the conditional probability that a given sample belongs to a class $y_{i}$:

(28) $$p(y_{i}|x)=\frac{p(x|y_{i})p(y_{i})}{p(x)}$$

where x represents the feature of the sample. When $p(y_{i}|x)$ is maximum, the corresponding class is the class of the given sample.

(5) K-nearest neighbor

KNN is a distance-based classification method [9]. For a given sample, The KNN first finds K samples closest to the given sample by calculating the distance between the given sample and the known sample, which are called neighbors. Finally, the sample is classified as the class with the most occurrences among K’s nearest neighbors [20]. The nearest neighbor of KNN is set to 5. For two samples x and y, the Euclidean distance is used to measure the distance between the samples:

(29) $$d(x,y)=\sqrt{\sum_{i=1}^{n}{\left(x_{i}-y_{i}\right)}^{2}}$$

(6) Convolutional neural network

**Figure 3:**
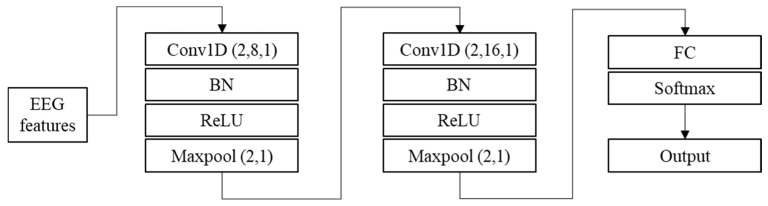
Structure of CNN.

CNN is a deep feed-forward neural network with a local connection and weight sharing, which is widely used in EEG classification [2]. CNN usually contains the input, convolutional, activation, pooling, fully connected, and output layers [14]. In this paper, a shallow CNN model is designed for EEG feature classification, and the model structure is shown in Figure 3.

(7) Long short-term memory

**Figure 4:**
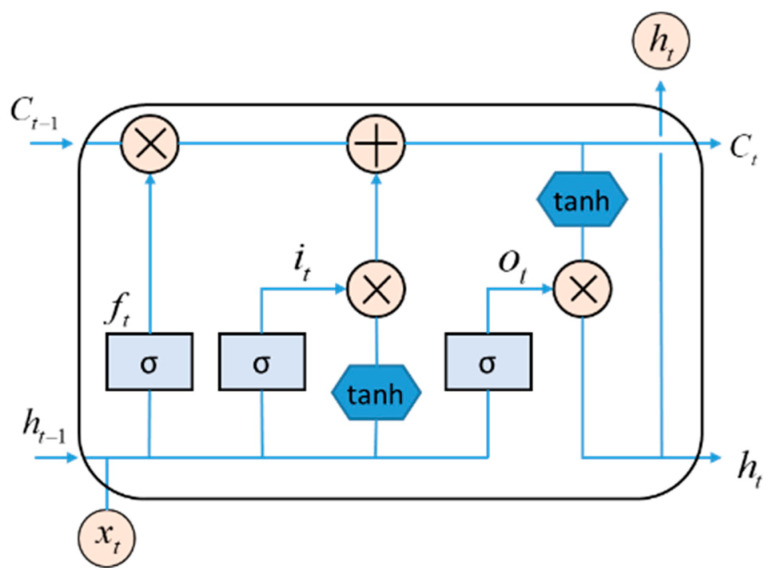
Structure of LSTM.

LSTM is a classical recurrent neural network that performs well in sequence data processing [18]. LSTM introduces the forgetting gate, input gate, and output gate based on traditional recurrent neural networks to control the flow of information, which can effectively solve the long-term dependence problem. The model structure is shown in Figure 4. The “hidden-size” and “num-layers” of LSTM in this paper are set to 20 and 1, respectively. The Bi-LSTM network consists of two independent LSTM layers that handle time series’ forward and reverse flow, respectively. The “hidden-size” and “num-layers” of Bi-LSTM in this paper are set to 10 and 1, respectively.

(8) Transformer encoder

**Figure 5:**
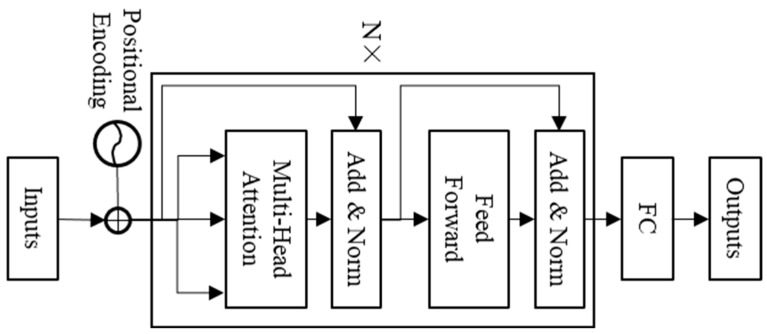
Structure of transformer encoder model.

The transformer is composed of an encoder and decoder and is a sequence model based on the self-attention mechanism [1]. This model is mainly used for various natural language processing tasks, such as translation and text generation, and is also gradually applied to time series prediction. This paper uses the transformer encoder (TE) to classify single-channel sleep EEG. The model structure is shown in Figure 5. The “nhead” and “num-layers” of TE in this paper are set to 3 and 3, respectively.

### 2.4. HMM-Based Correction

**Figure 6:**
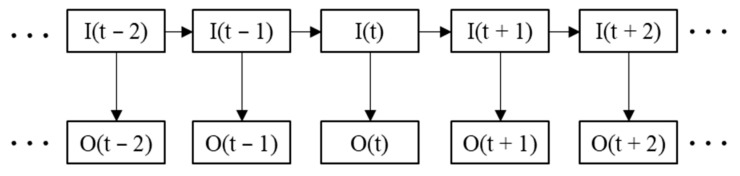
Structure of the HMM.

The transition of sleep states has an apparent time structure. That is, the sleep state of the next stage is related to the sleep state of the previous stage [17]. Therefore, it is possible to correct some of the misclassified sleep states by observing previous sleep states. However, manual recognition of misclassified sleep states is time-consuming and subjective. Some studies have pointed out that HMM can learn the sleep transition rule and realize the adaptive correction of misclassified sleep states. Therefore, HMM is used in this paper to self-correct the prediction results [31]. The structure of the HMM is shown in Figure 6. I represents the sequence of hidden states, and the corresponding set is Q. O represents the sequence of observed states, and the corresponding set is V. The hidden state sequence is not visible, and the observed state sequence is visible.

The HMM can be represented using a set of parameters λ={A,B,π} [15]. A is the hidden state probability matrix, representing the probability of the current hidden state moving to the next hidden state. B is the observation probability matrix, representing the probability distribution corresponding to different observation results under the current hidden state. π is the initial probability distribution of the hidden states.

(30) $$A_{i,j}=p(I(t+1)=q_{j}|I(t)=q_{i})$$

(31) $$B_{i,j}=p(O(t)=v_{i}|I(t)=q_{i})$$

The classifier’s output is defined as the observed state sequence of the HMM, and the actual sleep state transition sequence is defined as the hidden state sequence of the HMM. According to the sleep stage division rule, the set of hidden and observed states in this paper is defined as Q=V={W,N1,N2,N3,REM}. During the model’s training, the dataset is divided into three parts, namely the training set, the validation set, and the test set. The training set is used to train the classifier model, and then, the trained classifier is used for the validation set. The predicted sequence and the actual sleep state sequence on the validation set are taken as the observed state sequence and hidden state sequence of the HMM. Then, the hidden state transition probability matrix A and the observed probability matrix B of the HMM are calculated using the maximum likelihood estimation.

(32) $${\hat{A}}_{i,j}=\frac{W(I(t)=q_{i},I(t+1)=q_{j})}{\sum_{j=1}^{N}W(I(t)=q_{i},I(t+1)=q_{j})}$$

(33) $${\hat{B}}_{i,j}=\frac{W(I(t)=q_{i},O(t)=v_{j})}{\sum_{j=1}^{M}W(I(t)=q_{i},O(t)=v_{j})}$$

where $W(I(t)=q_{i},I(t+1)=q_{j})$ represents the number of times the hidden state sequence transitions from $I(t)=q_{i}$ to $I(t+1)=q_{j}$. $W(I(t)=q_{i},O(t)=v_{j})$ represents the number of times the observation state is $O(t)=v_{j}$ when the hidden state is $I(t)=q_{i}$. N and M represent the length of the hidden and observed state sets, respectively, with N=M=5 in this paper. Since the sleep process begins in an awake state, the initial probability distribution is set to π=[1,0,0,0,0]. Through the above methods, the HMM λ={A,B,π} is successfully constructed.

In the test phase, the feature of the test set is input into the trained classifier model, and the prediction result is obtained. Currently, the test set’s prediction result is the observed state sequence O, and the corresponding hidden state sequence I is the final prediction result. Then, for the trained HMM model λ={A,B,π} and the prediction result O, the final prediction result is:

(34) $$I=\underset{I}{\arg\max}(p(I|O,\lambda ))$$

In this paper, the Viterbi algorithm [32] is used to solve the most likely hidden state sequence.

## 3. Sleep EEG Dataset

The Sleep-EDF database expanded (Sleep-EDFX, 2013 version) published on PhysioNet was used for the experiment [33]. The dataset consisted of two subsets, and this paper used the sleep cassette. Twenty healthy subjects (age: 28.65 ± 8.65, 10 males) participated in the experiment. Subject 13 collected the EEG for one night, and the other subjects collected the EEG for two nights, totaling 39 EEG signals for the whole night. The experiment collected EEG signals of Fpz-Cz and Pz-Oz channels, and the sampling frequency was 100 HZ. In this paper, the EEG signal of the Fpz-Cz channel is used for experimental verification. A trained technician manually scores the corresponding sleep EEG (sleep pattern) according to the Rechtschaffen and Kales manuals. Finally, the technician labeled the sleep states at 30 s intervals according to the R&K rules: W, N1, N2, N3, N4, REM, MOVEMENT, and UNKNOWN.

**Figure 7:**
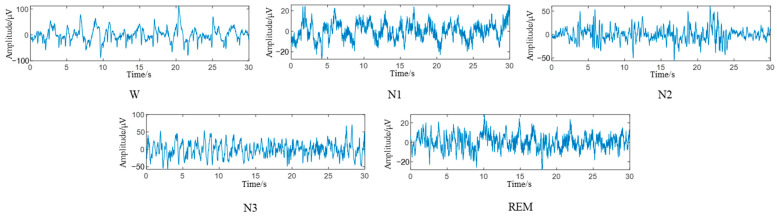
EEG at different sleep stages.

In the data preprocessing stage, a bandpass filter of 0.5–100 HZ is used to reduce the noise of the EEG. According to the AASM modified sleep classification criteria, we discard the MOVEMENT and UNKNOWN tags in the dataset. The N3 and N4 stages were merged into N3, and the sleep state was divided into five stages, namely W, N1, N2, N3, and REM. The EEG was segmented for 30 s, with 3000 sampling points per segment, and mapped to the labeled sleep stages. The 30 s EEG fragments corresponding to different sleep states are shown in Figure 7. Since nearly 24 h of EEGs were collected in the experiment, it was necessary to divide the night sleep time. In this paper, the EEG between staying awake for 30 min before falling asleep and staying awake for 30 min after waking up was intercepted, and the EEG of about 9 h was generally divided. The number of samples corresponding to each sleep stage in the dataset is shown in Table 3.

## 4. Results

### 4.1. Experimental Setup

In this paper, four-layer wavelet decomposition is designed to extract multi-domain EEG features, and the dimension of single-step time features is 1 × 105. We use the multi-step time feature matrix as the model’s input to enable the network to learn the sleep stage transition rules in the classification process. The dimension of the multi-step time feature matrix is T × 105, where T represents the step length. The time step designed in this paper is 3, so the dimension of the multi-step time feature matrix is 3 × 105. It should be noted that, for the traditional machine-learning model, the multi-step time feature matrix needs to be converted into a one-dimensional vector, so it is difficult for the traditional machine-learning model to learn the sleep transition rules during the classification process.

Ten-fold cross-validation was used for experimental verification. The 39-night EEG data were divided into ten pieces, one of which was taken as the test data each time, and the other nine were taken as the training data. During model training, 20% of the training data was divided into a validation set, which was used to verify model performance and build the HMM. The test set was used to test model performance and was not used during model training. The mean value of cross-validation was used as the model’s evaluation indicator. The accuracy (ACC), macro average F1 score (MF1), and Coenkappa coefficient (kappa) were used as evaluation indicators [19,20,21]. All algorithms were completed on MATLAB 2019 and an Intel(R) Core(TM) i7-9750H CPU, 2.6 GHz.

(35) $$P_{i}=\frac{T_{P}^{i}}{T_{P}^{i}+F_{P}^{i}}$$

(36) $$R_{i}=\frac{T_{P}^{i}}{T_{P}^{i}+F_{N}^{i}}$$

(37) $$F1_{i}=\frac{2P_{i}R_{i}}{P_{i}+R_{i}}$$

(38) $$ACC=\frac{1}{M}\sum_{i=1}^{n}T_{P}^{i}$$

(39) $$MF1=\frac{1}{n}\overset{n}{\underset{i=1}{\sum }}F1_{i}$$

(40) $$kappa=\frac{p_{0}-p_{c}}{1-p_{c}}$$

where $T_{P}$, $F_{P}$, and $F_{N}$ represent true positive, false positive, and false negative, respectively. $p_{0}$ represents the overall accuracy, M represents the total number of samples. n represents the number of categories, and $p_{c}$ represents the accidental consistency error.

### 4.2. Comparison of Different Decomposition Methods

First, the performance of WT, EMD, VMD, and SSA in EEG decomposition is analyzed. The relevant parameters of the algorithm are set as follows. The number of decomposition layers of WT is four, and the wavelet function is db4. The first six intrinsic mode functions (IMFs) in the EMD are selected for calculation in this paper. The number of decomposition layers for VMD and SSA is set to six to ensure that the number of IMFs is the same as that of EMDs.

**Figure 8:**
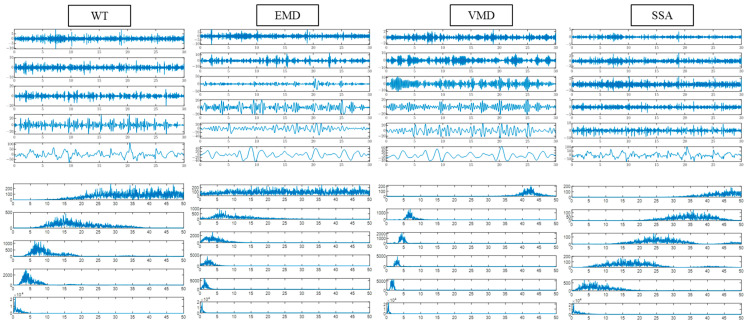
Component signal and spectral diagram of different signal decompositions.

WT, EMD, VMD, and SSA were used to decompose the first EEG fragment of subject 1, and the results are shown in Figure 8. All four methods can extract the low-frequency and high-frequency components of EEGs. The components of EMD and VMD are more concentrated in the frequency band below 10 HZ, and there is a lack of component information above 10 HZ. The components of WT and SSA are evenly distributed at 0–50 HZ, but the mode aliasing of SSA is severe. The results of signal decomposition show that WT can extract more comprehensive brain frequency band information, and the mode aliasing is not severe.

**Figure 9:**
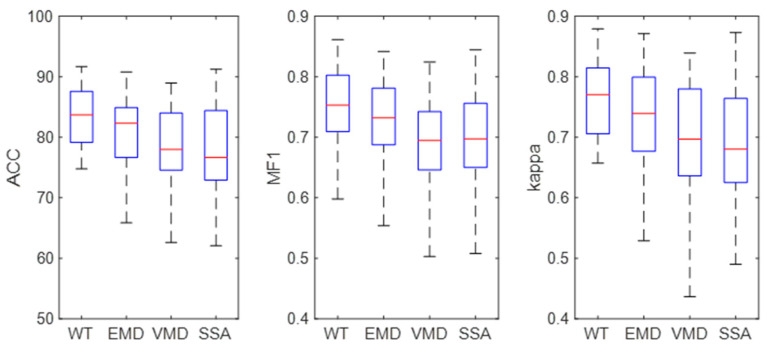
Classification results of different decomposition methods.

EEG features were extracted based on WT, EMD, VMD, and SSA, and then, the LSTM was used for EEG classification. The results are shown in Figure 9. It can be seen from the figure that the ACC, MF1, and kappa values of the WT-based method are 81.66%, 0.74, and 0.74, respectively, which are higher than that for other methods. The confidence interval of the WT-based method is lower than that of EMD, VMD, and SSA, indicating that the WT-based method is more stable in single-channel sleep EEG. The results show that WT is suitable for single-channel sleep EEG.

### 4.3. Classification Results of Different Classifiers

Different classifiers are used to classify the multi-step time features, and the results are shown in Table 4. It can be found from the table that the classification accuracies of LSTM, Bi-LSTM, and TE are higher than 81%, while the classification accuracies of other models are lower than 81%. The results show that, based on the multi-step time feature and timing model, the potential sleep transition rules can be learned during the classification process, which improves the classification accuracy.

**Figure 10:**
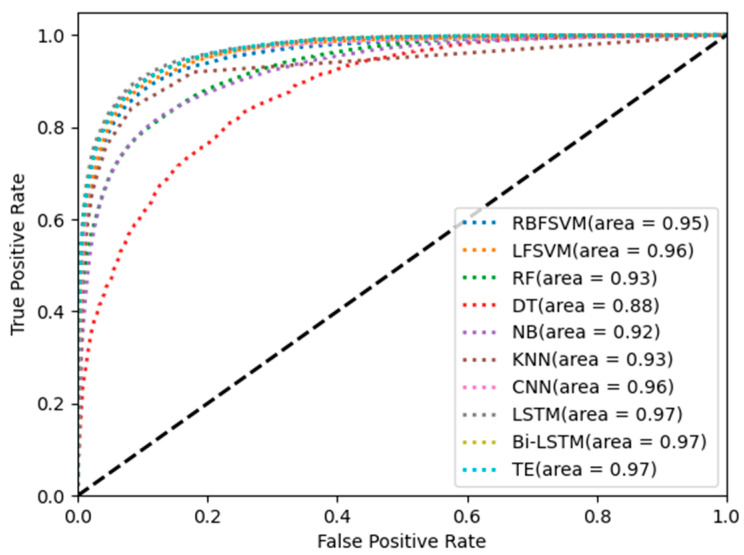
ROC curves of different methods.

Figure 10 shows the ROC curves of the different methods. The area under the ROC curve (AUC) of LSTM, Bi-LSTM, and TE is 0.97, which is higher than that of the other classifiers. The results indicate that the method based on multi-step time features and the time series network can improve single-channel sleep EEG classification accuracy.

**Figure 11:**
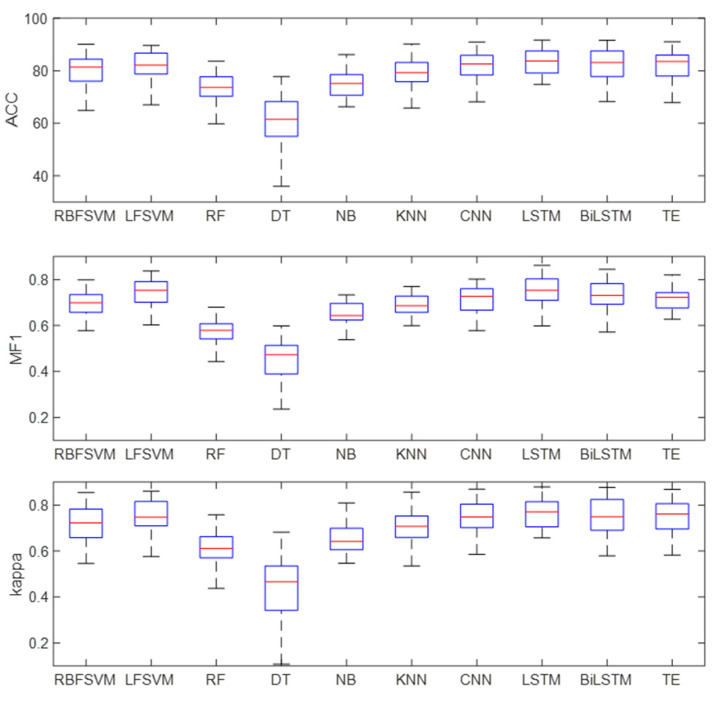
Classification results of different classifiers.

Figure 11 shows the box diagram of the results. It can be found from the figure that the upper and lower limits of DT differ greatly, indicating that DT has poor robustness. The upper and lower limits of the other methods have little difference, which suggests that these methods are robust. The median of accuracy, MF1, and kappa of LSTM are slightly higher than those of the other methods. Meanwhile, the lower limits of accuracy, MF1, and kappa of the LSTM are higher than those of the other methods, which indicates that the performance of the LSTM is superior to other methods.

### 4.4. Performance Analysis of HMM

After classification, the HMM is used to correct the classification results. To verify the effectiveness of HMM, the EEG classification results were compared before and after HMM processing, as shown in Table 5. It can be seen from the table that the ACC, MF1, and kappa improved after HMM processing. The LSTM performed best among the three classifiers; the classification accuracy, MF1, and kappa are 82.71%, 0.75 and 0.76, respectively. After HMM processing, the classification accuracy, MF1, and kappa of LSTM increased by 1.05%, 0.01, and 0.02, respectively, indicating that HMM can use the sleep transition rules to achieve adaptive correction of the classification results.

**Figure 12:**
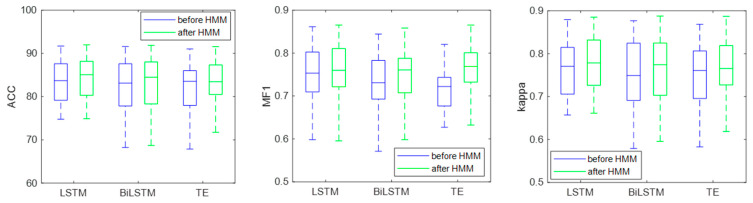
Classification results before and after HMM.

Figure 12 shows the box diagram of the classification results. It can be seen from the figure that, after HMM processing, the median values of ACC, MF1, and kappa increased, and the lower limit of MF1 and kappa of the Bi-LSTM and transformer also increased. The results show that the HMM can use sleep transition rules to improve the classification results and that it has good robustness.

**Figure 13:**
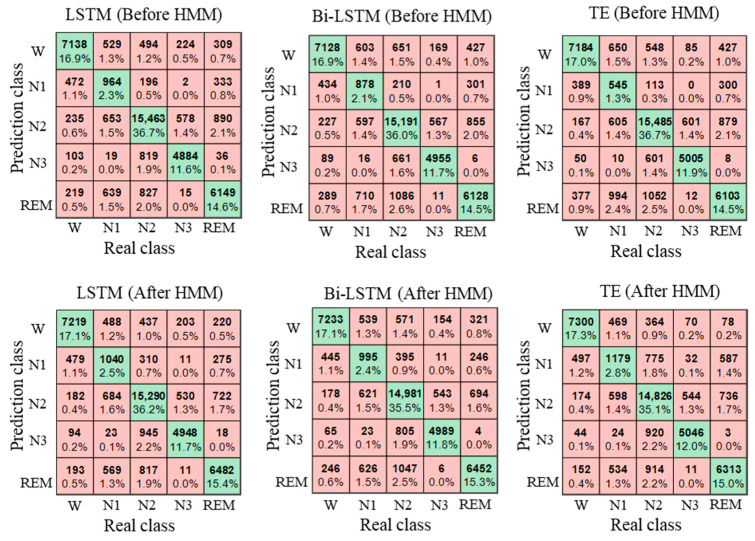
Confusion matrix of classification results.

Figure 13 shows the confusion matrix of the classification results. It can be found from the figure that there exists an imbalance in the data, in which the data number of N1 is about 2000, and the data number of other categories is greater than 5000. The number of correctly identified N1 is less than 50%, and the number of correctly identified remaining categories is more than 80%, which indicates that the classification accuracy of N1 is low. After HMM processing, the number of N1 correctly identified increased, which indicated that HMM was conducive to the classification of N1.

### 4.5. Effect of Wavelet Function on the Method

**Figure 14:**
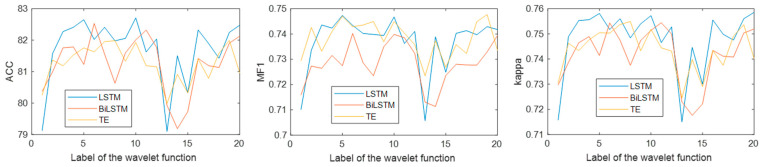
Classification results under different wavelet functions.

Figure 14 shows the classification results under different wavelet functions. Horizontal coordinates 1 through 20 represent {‘bior11’, ‘bior22’, ‘bior33’, ‘bior44’, ‘coif1’, ‘coif2’, ‘coif3’, ‘coif4’, ‘db2’, ‘db4’, ‘db6’, ‘db8’, ‘rbio11’, ‘rbio22’, ‘rbio33’, ‘rbio44’, ‘sym2’, ‘sym4’, ‘sym6’, ‘sym8’}. It can be seen from the figure that the LSTM performs better than the Bi-LSTM and transformer encoder on most wavelet functions. When the wavelet function is db4, the LSTM has the best performance, and the classification accuracy, MF1, and kappa are 82.71%, 0.75, and 0.76, respectively. The results show that LSTM can learn the sleep transition rules more accurately and is more helpful for single-channel sleep EEG classification.

### 4.6. Effect of the Multi-Step Time Features

**Figure 15:**
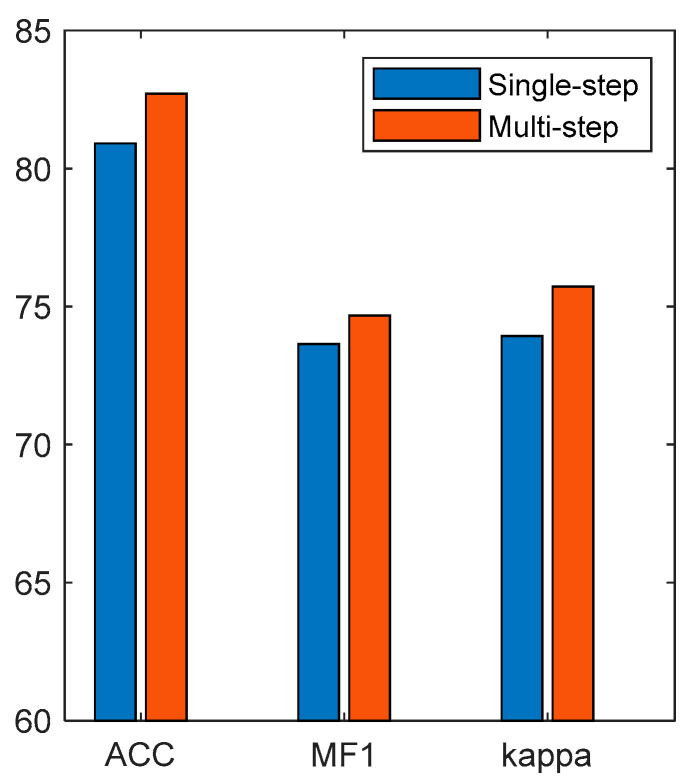
Classification results of single-step features and multi-step time features.

Figure 15 shows the classification results of single-step features and multi-step time features. It can be seen that, compared with single-step features, multi-step time features have higher classification accuracy, MF1, and kappa. Compared with the single-step-based method, the accuracy, MF1, and kappa of the multi-step-based method improved by 1.8%, 0.01, and 0.02, respectively. The results show that LSTM can learn the sleep transition rules in the multi-step time features and improve the accuracy of the EEG classification.

### 4.7. Comparison with Existing Research

Table 6 shows the classification results of the proposed method and the existing studies on Sleep-EDFx. Some methods [12,14] used deep networks to extract depth features from the EEG. Although most studies point out that depth features can extract more information than manual features [15], the results of this paper show that manual features are more competitive in the classification of sleep EEG. Some studies [17,19,34] and proposed methods first extract manual features from the EEG and then use machine learning to classify the sleep EEG. The proposed method improves the classification accuracy of sleep EEG from two aspects. First, WT is used to extract the deep information from the EEG. The second is to learn the sleep transition rules to improve classification accuracy. Based on the results, the proposed method is more competitive. The results show that the proposed method can achieve high-precision single-channel sleep EEG classification and is superior to most existing methods.

## 5. Discussion

This paper proposed a single-channel sleep EEG classification method based on the LSTM and HMM. First, the proposed method used signal decomposition and multi-domain feature extraction to obtain deep EEG information. Second, the proposed method learned the sleep transition rules in the classification process using multi-step time features and temporal networks. Third, the proposed method used HMM to post-process the classification results and realize the automatic correction of the classification results. A complete experiment was conducted on the Sleep-EDFx dataset. The results show that the proposed method can achieve high-precision single-channel sleep EEG classification and is superior to most existing methods.

The performance of four signal decomposition methods (WT, EMD, VMD, and SSA) in single-channel sleep EEG classification was compared. The results in Figure 8 show that WT achieved the highest accuracy, indicating that WT is more suitable for EEG decomposition. Then, the proposed method was used to classify single-channel sleep EEG, and the performance of 20 different wavelet functions was compared. The results in Figure 14 show that the classification method based on WT-db4 and LSTM had the best performance, and the accuracy, MF1, and kappa were 82.71%, 0.75, and 0.76, respectively. This paper discussed the performances of various signal decomposition methods and wavelet functions in single-channel EEG classification, providing a reference for other EEG analyses.

Table 4 and Figure 10 show the classification results of different classifiers in single-channel sleep EEG. The results show that the classification accuracy of temporal networks (LSTM, Bi-LSTM, and TE) in single-channel sleep EEG was higher than 81%, and the AUC was equal to 0.97, which was higher than that of other classifiers. The sleep state transition had potential regularity, and the temporal network could learn temporal information from temporal features so that the temporal network could improve the EEG classification accuracy. Figure 11 shows the box diagram of the prediction results, and the distribution state of the classification results can be observed. It can be seen from the figure that LSTM, Bi-LSTM, and TE had good robustness, and the minimum accuracy of LSTM was about 70%, indicating that the method had high robustness.

Table 5 and Figure 12 show the EEG classification results before and after HMM processing. Before HMM processing, the classification accuracy, MF1, and kappa of the LSTM were 81.66%, 0.74, and 0.74, respectively. After HMM processing, the classification accuracy, MF1, and kappa of LSTM were 82.71%, 0.75, and 0.76, respectively. The results show that the classification accuracy, MF1, and kappa improved after HMM processing, which indicated that the HMM could modify the classification results by learning the sleep transition rules after the classification was completed. Figure 13 shows the confusion matrix of the classification results, and the specific classification results of different sleep stages can be observed. As can be seen from the figure, the amount of the N1 stage was significantly less than that of the other stages, so the sample imbalance existed in the sleep EEG dataset, which also led to the low classification accuracy of the N1 stage. At the same time, after HMM processing, the number of N1 correctly identified increased, indicating that HMM can improve the sensitivity of the N1 stage.

Finally, we compared the performances of single-step features and multi-step temporal features in EEG classification, and the results are shown in Figure 15. The results show that the performance of the multi-step time features was better than that of the single-step features. The single-step features only used the single-moment EEG features as the model input, so it was difficult for the model to learn the sleep transition rules. The multi-step time features recombined the EEG features of multiple moments into a multi-step time feature matrix to contain the timing information of sleep transition, which was conducive for the timing network to learn the sleep transition rules.

There are also some limitations. First, the proposed method is challenging to directly apply to other EEG classifications of discontinuous states, such as motor-imaging EEG classification. Second, the direct application of the proposed method to the classification of a multi-channel EEG may result in dimensional disaster. Third, the classification accuracy of the proposed method in the N1 stage still needs to improve. Some aspects of the proposed method can be improved in future work so that the proposed method can be used for discontinuous state EEG and multi-channel EEG classification. At the same time, we can pay more attention to the EEG feature-extraction method of the N1 stage in the future.

## 6. Conclusions

This paper proposed a single-channel EEG classification method based on the LSTM and HMM, and a complete experiment was carried out on the Sleep-EDFx dataset. The performance of EMD, VMD, SSA, and WT in EEG classification was discussed in this paper. The results showed that WT was suitable for EEG decomposition. On this basis, the performance of 20 wavelet functions in EEG classification was discussed, which provided a reference for other EEG-related studies. During classification, the multi-step time features and LSTM were used to learn the sleep transition rules and improve the classification accuracy. After the classification, the proposed method used the HMM to learn the sleep transition rules and realize the adaptive correction of the classification results. The results showed that the proposed method successfully learned the sleep transition rules from two perspectives and significantly improved the classification accuracy of single-channel sleep EEG. The classification accuracy, MF1, and kappa of the proposed method were 82.71%, 0.75, and 0.76, respectively.

## Figures and Tables

**Table 1:** Specific information on wavelet function.

Class of WF	Wavelet Function
Daubechies	db2, db4, db6, db8
Symlets	sym2, sym4, sym6, sym8
Coiflets	coif1, coif2, coif3, coif4
Biorthogonal	bior1.1, bior2.2, bior3.3, bior4.4
ReverseBior	rbio1.1, rbio2.2, rbio3.3, rbio4.4

**Table 2:** Time domain, frequency domain, and nonlinear features.

Domain	Feature	Dimension
Time	Absolute mean, standard deviation, skewness, kurtosis Hjorth parameters (activity, mobility, complexity)	35
Frequency	Mean, standard deviation, skewness, kurtosis, mean square value Average power spectral density, power, and power ratio of different sub-bands	45
Nonlinear	Approximate entropy, differential entropy, Shannon entropy, CO complexity, fractal dimension	25

**Table 3:** Number of samples for each sleep stage.

Subjects	W	N1	N2	N3	REM	Sum
S0	371	117	623	517	340	1968
S1	319	201	1222	201	346	2289
S2	253	278	947	214	342	2034
S3	276	106	885	188	408	1863
S4	385	303	1134	147	466	2435
S5	430	158	833	249	248	1918
S6	335	146	824	265	289	1859
S7	531	173	795	384	366	2249
S8	467	107	591	632	391	2188
S9	289	100	1073	277	497	2236
S10	299	182	1278	31	406	2196
S11	252	31	898	240	309	1730
S12	466	169	750	187	457	2029
S13	155	57	497	147	172	1028
S14	368	56	790	296	446	1956
S15	979	88	792	355	500	2714
S16	395	97	907	293	455	2147
S17	865	65	1015	403	427	2775
S18	285	180	678	507	234	1884
S19	564	190	1267	170	618	2809
Sum	8284	2804	17,799	5703	7717	42,307

**Table 4:** Classification accuracy of different classifiers.

Method	ACC	MF1	Kappa
RBFSVM	79.14	0.69	0.71
LFSVM	79.92	0.73	0.72
RF	72.10	0.56	0.59
DT	60.35	0.45	0.44
NB	73.16	0.64	0.63
KNN	77.83	0.68	0.69
CNN	80.53	0.71	0.73
LSTM	81.66	0.74	0.74
Bi-LSTM	81.09	0.72	0.74
TE	81.21	0.71	0.74

**Table 5:** Classification results before and after HMM.

Classifier	Postprocessing	ACC	MF1	Kappa
LSTM	/	81.66	0.74	0.74
	HMM	82.71	0.75	0.76
BiLSTM	/	81.09	0.72	0.74
	HMM	82.01	0.74	0.75
TE	/	81.21	0.71	0.74
	HMM	81.93	0.74	0.75

**Table 6:** Results of the proposed method and existing methods on Sleep-EDFx.

Methods	Classifier	Channel	ACC	MF1	Kappa
[14]	Muti-task CNN	Fpz-Cz	81.9	0.74	0.74
[12]	CNN + Attention	Fpz-Cz	82.8	0.78	\
[19]	RF + HMM	Fpz-Cz	81.24	\	0.74
[17]	CNN + Bi-LSTM	Fpz-Cz	82	0.77	0.76
[34]	auto-encoder	Fpz-Cz	74.8	0.7	\
Proposed method	LSTM + HMM	Fpz-Cz	82.71	0.75	0.76

## Data Availability

All data files are available from https://physionet.org/content/sleep-edfx (accessed on 11 October 2023).
